# Eosinopenia in Patients With Typhoid Fever: A Case-Control Study

**DOI:** 10.7759/cureus.10359

**Published:** 2020-09-10

**Authors:** Uzma Ishaq, Jahanzeb Malik, Maliha Asif, Hina Zaib, Iqbal Haider, Tayyaba Zahid, Rana Abdul Sattar, Umar Ikram

**Affiliations:** 1 Hematology and Medical Oncology, Fauji Foundation Hospital, Rawalpindi, PAK; 2 Cardiology, Rawalpindi Institute of Cardiology, Rawalpindi, PAK; 3 Pathology, Rahber Medical and Dental College, Lahore, PAK; 4 Hematology, Ali Medical Center, Rawalpindi, PAK

**Keywords:** typhoid fever, eosinopenia, leukopenia, salmonella typhi

## Abstract

Background and objectives

Salmonella typhi is involved in one of the most prevalent infectious diseases of South East Asia, typhoid fever, but diagnostic tests cannot be performed regularly in developing countries. The objective of the study is to determine the association of eosinopenia with culture or serology-proven typhoid fever in patients, to determine the association of leukopenia with culture or serology-proven typhoid fever in patients and to determine independent predictors of eosinopenia.

Methods

This case-control study, done at Fauji Foundation Hospital, Pakistan, included patients with symptoms suggestive of typhoid fever, culture, or serology positive for typhoid fever and excluded patients who had been previously diagnosed with malaria and dengue hemorrhagic fever. After flagging cultures and serology, the records were viewed for leukocyte counts. Data, including age, gender, and clinical symptoms, were also recorded for analysis.

Results

Out of 200 participants, 59 participants with diagnosed typhoid fever had eosinopenia. There were 29 participants who had been diagnosed with typhoid fever via culture or serology and had leukopenia. Eosinopenia and leukopenia were more likely to be present in patients with a diagnosis of typhoid (OR: 9.60, 20.00). The independent predictors for eosinopenia were DOT enzyme immunoassay (DOT EIA) positive for Salmonella typhi, culture positive for Salmonella typhi and leukopenia (p<0.05).

Conclusion

The presence of eosinopenia and features or serology suggestive of typhoid would most likely be associated with cultures being positive and, therefore, might provide an efficient means to begin treatment.

## Introduction

Typhoid is caused by Salmonella typhi, which are gram-negative rods [1]. It is mainly transmitted via food or water reservoirs of infection contaminated by human excretions [2]. According to the World Health Organization (WHO), around 21 million people are infected worldwide each year with death tolls reaching up to 200,000 [3]. A higher incidence is in the Tropics and Southeast Asia where it mainly affects young adults and children [4]. There are multiple factors, including residence in rural areas and unhygienic water supplies, which contribute to the risk [5]. The variability in clinical presentation, with patients presenting with prolonged fever, frontal headache, malaise, and, in severe circumstances, intestinal perforation and neurological complications, requires a high index of suspicion for prompt diagnosis [6]. Despite culture being the gold standard for diagnosis, complete blood count (CBC) is the preferred test in resource-limited countries where reactive changes in leukocyte counts are deemed clues for the diagnosis of typhoid.

Although considered a key feature of typhoid, leukopenia is only present in 25% of the cases [7]. Differential leukocyte counts show consistent eosinopenia (80%) in several studies [8-10]. This gives a new dimension to the diagnostic approach in developing countries where results with a quicker yield are favored. The diagnostic yield is also important, for example, by using blood culture, there are 40% chances that the cultures will be positive after a span of three to seven days [11]. The importance of a quicker, diagnostic test in Pakistan is significantly increased because the emergence of the disease is increasing. In one of the studies conducted in Hyderabad, 486 cases had been identified from three major hospitals of the region and all of these cases were resistant to ceftriaxone using culture yield, but the study did not comment regarding white blood counts [12]. In another study, the number of Pakistani patients was found to be as high as 5,372 [13].

The objectives of the study were to determine the association of eosinopenia with culture or serology-proven typhoid fever in patients, to determine the association of leukopenia with culture or serology-proven typhoid fever in patients, and to determine independent predictors of eosinopenia.

## Materials and methods

This retrospective case-control study was conducted at the Fauji Foundation Hospital, Rawalpindi, Pakistan. The study period was six months. The study was conducted after permission from the Institutional Review Board and Ethics Committee of Fauji Foundation Hospital (IRB#125-18). The sample size was calculated using Open Source Epidemiologic Statistics for Public Health software. The sample was calculated as 206 patients; 103 cases and 103 controls using significance levels of 95%, a power of 0.80, a ratio of 1.0 between exposed and unexposed patients, with 38% of unexposed patients with the outcome and 58% of exposed patients with the outcome. The percentages regarding prevalence slightly varied after a preliminary view of the data records.

The inclusion criteria for the study were patients presenting with suggestive symptoms of typhoid, such as prolonged fever, nausea, vomiting, and diarrhea, and patients with blood culture identifying Salmonella typhi or serology sent for Salmonella typhi. The exclusion criteria were patients less than 13 years of age because the study did not aim to investigate pediatric age groups, patients with previously known diagnoses of malaria, and dengue hemorrhagic fever. The diagnosis of dengue hemorrhagic fever is based on the presence of thrombocytopenia and polymerase chain reaction positive for the species. The diagnosis of malaria is based on thick and thin-film based microscopy to determine the pathogen load and species respectively. Therefore, the cases were defined as patients with either symptomatic culture-proven typhoid fever or serology-proven typhoid fever. The controls included patients who met our inclusion criteria of suggestive symptoms, such as fever and other gastrointestinal symptoms, and did not have a culture or serology-proven typhoid fever.

For blood culture, the blood specimens (5 ml) were collected in the Brain Heart Infusion (BHI) culture bottle using an aseptic technique. Subculture was done on agar plates after 24 hours in samples showing growth. After Salmonella produced colorless colonies on the MacConkey agar plate, confirmation and species identification were done by biochemical and serological tests. The identification details were recorded on a separate Microsoft Excel (Microsoft Corporation, Redmond, Washington) spreadsheet. The data records were viewed for white blood cell (WBC) counts after serology and culture studies had been flagged as positive for patients suggestive of typhoid fever. The records were also viewed similarly for the control group. These white blood cell counts had been obtained using blood specimens (2 ml) in complete blood picture (CP) bottles containing ethylenediaminetetraacetic-acid (EDTA) as an anticoagulant. It had been analyzed on an automated hematology analyzer Sysmex XN 2000i system (Sysmex America, Inc., Illinois) for automated total leukocyte count (TLC) and automated absolute eosinophil count (AEC).

The primary outcome was defined as the presence of eosinopenia (absolute eosinophil count less than 0.40 X 109/L) in patients with blood cultures or serology positive for Salmonella typhi. The secondary outcome was defined as the presence of leukopenia (total white blood count less than 4.00 X 109/L) in patients with blood cultures or serology positive for Salmonella typhi. A few serology tests were also conducted to confirm the diagnosis of Salmonella typhi. These tests are usually considered as a part of the protocol of the center’s workup for the organism. The serology was considered positive if Salmonella typhi O or H titer was greater than 1:160. Immunoglobulin M (IgM) antibodies were also quantified in the serological analysis. Last, we also aimed to determine the independent predictors of eosinopenia.

Statistical analysis was performed using IBM's Statistical Package for the Social Sciences (SPSS) version 21 (IBM, Armonk, New York). For analysis, the participants were divided into two groups with and without eosinopenia. Qualitative variables were analyzed using descriptive statistics. Mean and standard deviation was calculated for quantitative variables, which include age and total leukocyte count. Age and leukocyte counts of both groups were compared using an independent t-test. Chi-square was applied to determine if the differences in symptoms, gender, and serology were significant. A p-value of less than 0.05 was considered significant. Contingency analysis was also performed for the presence of eosinopenia and leukopenia. A logistic regression analysis was performed to determine the independent predictors for eosinopenia.

## Results

There were 200 participants in the study after six patients with incomplete investigations had been excluded. There were 100 patients with culture or serology-proven typhoid fever and 100 patients with symptoms suggestive of typhoid fever but not proven by either culture or serology. There were 99 (49.5%) male and 101 (50.5%) female participants in the study. The mean age of the participants in the study was 32.04±16.30 years. The mean total leukocyte count was 7.20±3.50 X 109/L. The mean absolute eosinophil count was 0.31±0.20 X 109/L. There were 72 participants (35.0%) who had eosinopenia and 31 participants (15.5%) who had leukopenia. The clinical characteristics of the eosinopenic and non-eosinopenic groups are shown in Table 1.

**Table 1:** Clinical characteristics of both groups n: frequency, S.D.: standard deviation, DOT EIA: DOT enzyme immunoassay

Characteristics	Eosinopenia present	Eosinopenia absent	p-value
Age (Mean±S.D)	26.38±14.83	35.23±16.26	0.00
Gender (n, %)			0.11
Male	41 (56.94)	58 (45.31)	
Female	31 (43.06)	70 (54.69)	
Symptoms (n, %)			0.31
Fever ≥7 days	28 (38.89)	60 (46.88)	
Abdominal pain	15 (20.83)	24 (18.75)	
Nausea	9 (12.50)	12 (9.38)	
Vomiting	7 (9.72)	11 (8.59)	
Chills	7 (9.72)	6 (4.69)	
Diarrhea	5 (6.94)	6 (4.69)	
Constipation	1 (1.40)	4 (3.13)	
Headache	0 (0.00)	5 (3.89)	
Laboratory tests			
Total leukocyte count (X10^9^/L); (Mean±S.D.)	5.67±3.54	8.06±3.18	0.00
Leukopenia (n, %)	24 (33.33)	7 (5.47)	0.00
DOT EIA positive (n, %)	39 (54.17)	102 (79.69)	0.00
Widal test positive (n, %)	6 (8.33)	19 (14.84)	0.18
Culture positive for Salmonella typhi (n, %)	59 (81.94)	41 (32.03)	0.00

Table 2 shows results regarding the presence of eosinopenia in patients with and without typhoid fever. It was seen that eosinopenia was 9.63 times (CI: 4.75-19.51) more likely to be present in patients with either culture or serology-proven typhoid fever as compared to patients without culture or serology-proven typhoid fever (p=0.00).

**Table 2:** Eosinopenia in cases and controls

Exposure	Outcome		Total number of patients
	Eosinopenia present	Eosinopenia absent	
Typhoid fever present	59	41	100
Typhoid fever absent	13	87	100
Total number of patients	72	128	200

A similar analysis was done for leukopenia (Table 3). The cases and controls were divided into two groups; with and without leukopenia. It was noted that leukopenia was 20.00 times (CI: 4.62-86.62) more likely to be present in patients with either culture or serology-proven typhoid fever as compared to patients without culture or serology-proven typhoid fever (p=0.00).

**Table 3:** Leukopenia in cases and controls

Exposure	Outcome		Total number of patients
	Leukopenia present	Leukopenia absent	
Typhoid fever present	29	71	100
Typhoid fever absent	2	98	100
Total number of patients	31	169	200

A logistic regression analysis was performed to determine other predictors of the study. The presence of eosinopenia was the dependent variable for the model. The results are shown in Table 4.

**Table 4:** Logistic regression analysis CI: confidence interval; DOT EIA: DOT enzyme immunoassay

Variables	Unadjusted estimates			Adjusted estimates		
	Risk	95% CI	p-value	Risk	95% CI	p-value
Age	0.96	0.90-1.03	0.28	1.01	0.98-1.04	0.41
Gender	0.79	0.13-4.83	0.80	0.45	0.20-1.04	0.06
Symptoms	0.01	0.00-0.73	0.24	0.02	0.00-0.95	0.29
Total leukocyte count	0.37	0.15-0.93	0.03	0.94	0.81-1.11	0.51
Presence of leukopenia	1.62	0.14-18.37	0.69	0.16	0.04-0.67	0.01
DOT EIA positive	0.68	0.09-5.22	0.71	3.64	1.22-10.91	0.02
Widal test positive	1.17	0.07-20.63	0.92	1.65	0.36-7.45	0.52
Culture positive for Salmonella typhi	3.24	0.43-24.53	0.26	8.32	3.24-21.38	0.00

After adjusting the estimates, the independent predictors of eosinopenia were leukopenia, DOT EIA, and culture-positive for Salmonella typhi.

## Discussion

In our study, there were 99 male and 101 female participants. This result is relatively similar to another study in which 50.7% of the participants were women [13]. The mean age of the participants in the study was 32.04±16.30 years. Our population was relatively younger when compared to participants from a 2019 study who had a mean age of 33.10±6.50 years [13]. This implies that in non-pediatric age groups, the age of presentation might vary depending on geographical factors.

There were 59 participants with a diagnosis of typhoid fever by either serology or culture who had eosinopenia as compared to 13 participants with eosinopenia without typhoid fever. This proportion was relatively lower when compared to another study in which 82.1% of patients had absolute eosinopenia and positive blood cultures [14]. However, in our study, the diagnosis of typhoid was not limited to blood cultures and included serology as well. Leukopenia was present in 29 participants with a diagnosis of typhoid and two participants without a diagnosis of typhoid. This proportion was relatively higher when compared to a study in which 30.8% of the participants had leukopenia [14].

The relationship between acute infection and eosinopenia has been previously described. Sequestration of eosinophils during margination causes certain cytokines to be released which leads to decreased eosinophil counts [15]. Eosinopenia has been described for other bacterial febrile illnesses, such as pneumonia (9.9%), pyelonephritis (26.2%), prostatitis (8.4%), appendicitis (26.2%), cholecystitis (8.4%), and diverticular sigmoiditis (5%), as a general inflammatory response [16]. In comparison, eosinopenia was present in 13 participants without diagnosis suggestive of typhoid fever.

Our study clearly shows an association of leukopenia and eosinopenia with enteric fever with an odds ratio of 20.00 and 9.60, respectively. The odds ratio for leukopenia was comparatively higher when compared to a study investigating markers for the prompt diagnosis of typhoid fever, including leukopenia (OR: 11.8) [17]. However, in contrast to our study, it was noted that neutropenia was also another important marker. In contrast to our study, eosinopenia has been noted in patients with enteric fever (OR: 1.77) and not typhoid fever as reported in our study [18].

One of the independent predictors of eosinopenia was serology, i.e., DOT EIA, positive. This result was partly supported by the findings of another study that mentioned the sensitivity, specificity, and positive and negative predictive value of the test to be 91.42%, 90.00%, 88.88%, and 92.30%, respectively [19]. However, the serology had not been reported as a predictor in earlier literature and thought of as an alternative to the Widal test. Another predictor of eosinopenia was culture positivity for Salmonella typhi. This result provides important information because complete blood counts usually take lesser time as compared to cultures. While eosinopenia has been reported in patients with cultures positive for typhoid, it can be assumed from the results that in patients with eosinopenia and positive serology, there is a likely chance that cultures for Salmonella typhi would be positive.

Our study has a few limitations. The study was limited to one center. The study did not aim to investigate a pediatric group which could have presented a new dimension to the findings. The travel history of the patients was not explored in depth. The white blood cell counts could not be tracked over a specified period because of administrative difficulties at the center. Despite these limitations, eosinopenia is more commonly found in patients with typhoid fever and can, therefore, be used in cases with high clinical suspicion.

## Conclusions

The presence of eosinopenia in patients with features or serology suggestive of typhoid fever would most likely be associated with cultures being positive and, therefore, might provide an efficient means to begin treatment. In endemic areas, where the patient load is high, the finding can guide further treatment and expedite referrals to specialized centers.
